# Observations on the Cause of Paralysis in Painter's Colic

**Published:** 1828-10

**Authors:** George Kitson


### PATHOLOGY OF PARALYSIS.

### *Observations on the Cause of Paralysis in Painter's Colic.*

By George Kitson, Esq.

Several cases having occurred during the last five years which gave me reason to suppose that the cause of the paralysis from which painters, and others who are much in the habit of using lead, are so liable to suffer, might be found in the spinal canal, I was induced to examine a man who died under somewhat peculiar circumstances in the hospital; and the result of the examination having in some measure justified my anticipation, I am induced to send the case for insertion in the London Medical and Physical Journal, should it be deemed of sufficient importance to merit public attention. There are at all times numerous instances of this disease in the Bath Hospital; but so rarely does it prove fatal, that there has not been one instance of examination after death during eleven years that I have been surgeon to that institution.

John Mastin, a painter, aged forty, was admitted into the Bath Hospital, July 11th, 1828. The case sent to the hospital, which was most imperfectly stated, represented him to be suffering from rheumatic pains, chiefly of the hands and arms, without fever.

Mr. Bush, the house apothecary, collected from him that he had had three attacks of "dropt hands;" that the last, which occurred about three months since, was the most severe, and was preceded by colic. He had been at sea, and had lived freely; his mind appeared somewhat confused, and the pupils more than naturally dilated.

Dr. Barlow saw him the same day, when he found not rheumatism, but "dropt hands," from the poison of lead. The man made no complaint whatever, but his tongue being white and furred, the Doctor ordered him cathartic pills and an antimonial saline.

On the 13th, Dr. Barlow was called to see him. He complained of acute pain across the chest, with some pain of head. The pulse was ninety-six, full, hard, bounding; the tongue parched.

He was ordered to be bled; the saline every three hours, and cathartic pills.

14th.—The blood was buffed and cupped. He expressed himself much relieved. Pulse softer, and tongue moist.

16th.—In consequence of some pain of head, Mr. Bush applied leeches to the temples.

17th.—Some relief from the leeches. Appearances indicated that disease was going on within the head.

He was ordered to be cupped at the nape of the neck, and a blister to be applied.

18th.—Said he was rather better. Though able to answer questions distinctly, there was some confusion of mind.

19th.—He seemed better. Pulse ninety; tongue moist; bowels open.

20th.—A tendency to coma.

The head was shaved, and cold applied to it during the day, from its appearing to soothe. At night, a blister to the vertex.

21st.—Pulse seventy-two. Intelligent, apparently, in answering questions; but mind confused, and obviously much disturbance within the head.

Calomel gr. ij. quartis horis.

22d.—Died early in the morning.

N.B. Sensation was not defective; nor was there any paralytic affection of the lower extremities.

On examining the body, nine hours after death, a very considerable quantity of dark-coloured fluid was found between the dura mater and arachnoid membrane, in the cranium, and spinal canal. Colourless fluid was also effused between the arachnoid and pia mater. The brain was extraordinarily firm; the cortical substance pale; and, from the state of the pia mater, and the spots of blood exhibited on dividing the medullary substance, there was not any reason to suppose inflammation had existed. No fluid was found in the ventricles. The theca vertebralis was distended with bloody fluid. The pia mater covering the cord in its passage through the inferior and superior dorsal vertebræ was injected with blood; so much so that for about three inches on the anterior surface the membrane was uniformly of a bright red colour: posteriorly, the membrane was less vascular, as well as less firmly attached to the cord. The superior part of the pia mater had the usual appearance, and it gradually approached the natural state as it descended to the lumbar region.

It appears probable, from the foregoing statement, that the disease commenced in the superior part of the spinal canal; and, as the nerves forming the axillary plexus are given off at the part most materially diseased, it is fair to infer that it was the cause of the paralytic affection of the arms. The serous effusion which produced the confusion of mind, and ultimately proved fatal, in all probability did not take place until a few days previous to the man's dissolution; and as it may be doubted whether there be any evidence of actual inflammation of the brain or its membranes having existed, it is not improbable that the effused fluid was produced by the disease in the spinal canal.

*Bath; August 15th,* 1828.

